# Study on the Antifungal Activity of Gallic Acid and Its Azole Derivatives against *Fusarium graminearum*

**DOI:** 10.3390/molecules29091996

**Published:** 2024-04-26

**Authors:** Yilin Zheng, Yuqi Geng, Wenlong Hou, Zhe Li, Caihong Cheng, Xiuping Wang, Yuedong Yang

**Affiliations:** 1 Hebei Key Laboratory of Active Components and Functions in Natural Products, Hebei Normal University of Science and Technology, Qinhuangdao 066004, China; 17333546871@163.com (Y.Z.); 17531372375@163.com (Y.G.); wenlonghou@126.com (W.H.); kycyyd@126.com (Y.Y.); 2 College of Chemical Engineering, Hebei Normal University of Science and Technology, Qinhuangdao 066004, China; 3 Analysis and Testing Center, Hebei Normal University of Science and Technology, Qinhuangdao 066004, China; wangxiuping0721@163.com; 4 Hebei Key Laboratory of Crop Stress Biology, College of Agronomy and Biotechnology, Hebei Normal University of Science and Technology, Qinhuangdao 066000, China; zheli2007@163.com

**Keywords:** gallic acid, azoles, *Fusarium graminearum*, antifungal activity, density functional theory

## Abstract

The wheat scab caused by *Fusarium graminearum* (*F. graminearum*) has seriously affected the yield and quality of wheat in China. In this study, gallic acid (GA), a natural polyphenol, was used to synthesize three azole-modified gallic acid derivatives (AGAs1–3). The antifungal activity of GA and its derivatives against *F. graminearum* was studied through mycelial growth rate experiments and field efficacy experiments. The results of the mycelial growth rate test showed that the EC_50_ of AGAs–2 was 0.49 mg/mL, and that of AGAs–3 was 0.42 mg/mL. The biological activity of AGAs–3 on *F. graminearum* is significantly better than that of GA. The results of field efficacy tests showed that AGAs–2 and AGAs–3 significantly reduced the incidence rate and disease index of wheat scab, and the control effect reached 68.86% and 72.11%, respectively. In addition, preliminary investigation was performed on the possible interaction between AGAs–3 and *F. graminearum* using density functional theory (DFT). These results indicate that compound AGAs–3, because of its characteristic of imidazolium salts, has potential for use as a green and environmentally friendly plant-derived antifungal agent for plant pathogenic fungi.

## 1. Introduction

Wheat is one of the important global food crops and is susceptible to various pests and pathogens. *Fusarium graminearum* (*F. graminearum*) is one of the most serious pathogens, causing a serious decrease in wheat yield and quality due to Fusarium head blight (FHB or wheat scab) [1,2]. Moreover, the various secondary metabolites produced by *F. graminearum* during wheat infection can increase the risk of cancer in humans and animals [3]. Chemical control is one of the main measures to prevent and control wheat scab. However, the pathogen of wheat scab has developed resistance to various pesticides because of the long-term and large-scale use of chemical drugs, leading to a decrease in control effectiveness and causing environmental pollution and food security issues [4]. Therefore, finding efficient, broad-spectrum, low-cost, and environmentally friendly antifungal compounds is an urgent issue for technical personnel in this field.

Natural products, with advantages such as biocompatibility, structural diversity, and unique mechanisms of action, are considered an important source for developing new fungicides [5,6]. Gallic acid (GA) is a natural polyphenolic compound found in various plants, such as chestnuts, gallnuts, sumac trees, witch hazels, watercress, oak bark, tea, betel nuts, and blackberries [7], with a widely and easily obtainable source. Various pharmaceutical and chemical industries have expressed interest in GA due to its biological activities and commercial applications such as antioxidant, anticancer, antibacterial, antiviral, anti-inflammatory, etc. [8,9,10,11,12,13,14]. Thus, due to its abundance in nature and diversity of bioactivity, GA is considered an important molecule with significant development prospects for designing new and effective drugs. In the past decades of their development, GA derivatives have shown various excellent biological activities [15,16]. For example, the alkyl and arylgallates of GA exhibit high biological activity attributed to their long chains, which may help to improve cell permeability and lipid or hydrophobic pockets in target molecules [17]. Other derivatives with various activities have been investigated one after another, such as linear and branched gallic acid containing an amide moiety, gallic acid hydrazides, galloyl-heterocyclic hybrids, peptide-based hybrids, sugar-based hybrids, etc. [10,18,19,20,21,22].

**Figure 1:**
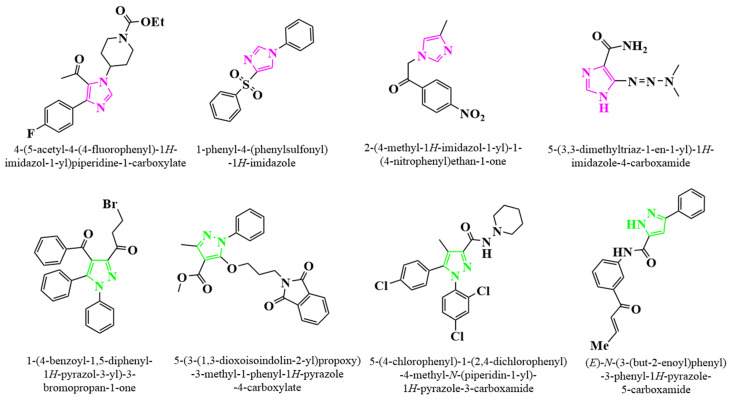
The structures of imidazole and pyrazole derivatives with various biological activities.

Azoles are a large class of five-membered heterocycles containing one or more nitrogen atoms, with or without other heteroatoms, and have always been considered as the preferred scaffold for designing novel therapeutic drugs [23]. Various nitrogen-containing and oxygen-containing azole compounds with aromatic and electron rich properties, such as imidazole, pyrazole, triazole, tetrazole, thiazole, etc., have been widely studied due to their diverse biological activities [24,25]. Previous studies have shown that azole derivatives, especially for pyrazole and imidazole derivatives (structures shown in Figure 1), have various biological activities, such as antifungal, antibacterial, anticancer, anti-inflammatory, and antituberculosis activities [26,27,28,29,30,31,32,33,34,35]. Xu et al. synthesized a series of aroyl pyrrolidine derivatives and investigated their in vitro inhibitory activity and in vivo anti-tumor activity against gelatinase (MMP-2, -9), of which most inhibitors exhibit good inhibitory activity (inhibition rate > 35%) [36]. Moreover, acyl pyrazoline derivatives also exhibit significant inhibitory effects on LPS stimulated NO generation and can effectively clear DPPH free radicals [37]. In addition, quaternary ammonium salts (QAS), with their unique structure, broad-spectrum antibacterial properties, low toxicity, good water solubility, and easy synthesis, are widely used in various fields such as agricultural fungicides, public place disinfection, and water treatment [38,39].

Herein, taking into account the above aspects, this study used natural polyphenol GA as the lead compound, introduced imidazole and pyrazole into GA, and quaternized them to synthesize a series of azole-modified gallic acid derivatives (AGAs) to exert their synergistic biological activity. Then, the antifungal effects of these derivatives against the wheat Fusarium head blight pathogen, *F. graminearum*, was investigated both in vitro and in vivo, and the interaction between drugs and receptors was given a preliminary explanation through computational chemistry methods.

## 2. Results and Discussion

### 2.1. Structural Characterization of GA and Its Derivatives

**Figure 2:**
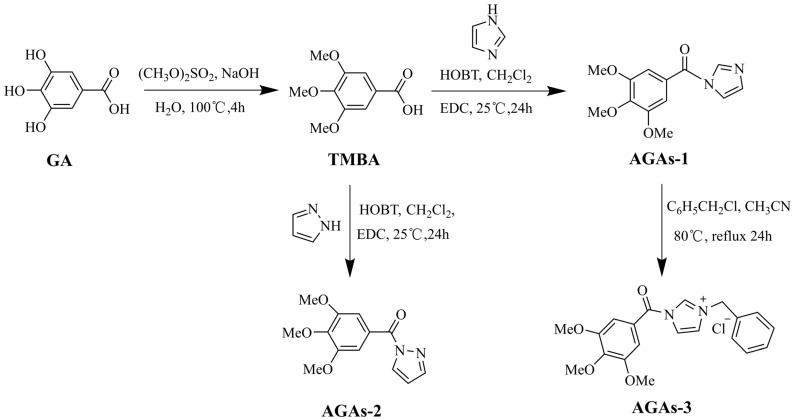
Synthesis routes of TMBA and azole-modified GA derivatives AGA1–3.

GA was obtained from the discarded chestnut leaves. To synthesize AGAs1–3, the intermediate 3,4,5-trimethoxybenzoic acid (TMBA) needed to be synthesized. In this experiment, GA was used as the starting material to first synthesize TMBA, and then AGAs1–2 were synthesized through the one-pot synthesis method using Im and Py as modified functional groups, respectively, as shown in Figure 2. AGAs–3 was prepared by heating and refluxing AGAs–2 with benzyl chloride. The post-treatment of this reaction is simple, and the yield is almost 100%. The structures of compounds GA, TMBA, and AGAs1–3 were characterized by nuclear magnetic resonance spectroscopy (NMR) and the ^1^H NMR and ^13^C NMR signal peaks of the characteristic functional groups of compounds were analyzed and assigned. This can be a mutual corroboration with the results of high-resolution mass spectrometry (HR-ESI(±)-MS). The NMR spectrum and HR-ESI(±)-MS are supplied in the supporting materials.

### 2.2. Inhibitory Effects of GA and Its Azole Derivatives on the Mycelial Growth Rate and Spore Germination of F. graminearum In Vitro

**Figure 3:**
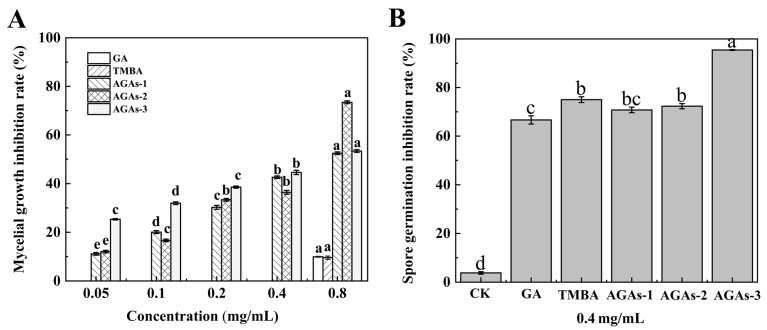
Effects of five compounds GA, TMBA, AGAs1–3 on different concentrations at 28 ± 1 °C. The inhibitory effect of *F. graminearum* mycelium growth (**A**); and the inhibitory rates of five compounds GA, TMBA, and AGAs1–3 on spore germination at 28 ± 1 °C for 5 h (**B**). The error bars represent the standard error (N = 3), and different lowercase letters indicate significant differences between treatments (*p* < 0.05).

The inhibitory effects of five compounds (GA, TMBA, and AGAs1–3) on the mycelial growth of *F. graminearum* at different concentrations are shown in Figure 3A. The azole-modified GA compounds AGAs1–3 had a significant inhibitory effect on the mycelial growth of *F. graminearum*. The inhibitory rate of compounds AGAs1–3 on the mycelial growth of *F. graminearum* was 11.00–73.46% in the concentration range of 0.05–0.8 mg/mL. Among the AGAs compounds, AGAs–3 had the highest inhibition rate on the growth of *F. graminearum* hyphae at a concentration of 0.8 mg/mL, reaching 73.46%. However, the inhibitory rates of compounds GA and TMBA on the mycelial growth of *F. graminearum* were both zero at concentrations of 0.05–0.4 mg/mL. When the concentration was 0.8 mg/mL, the inhibitory rates on the mycelial growth of *F. graminearum* were 9.93% and 9.55%, respectively. It can be seen that at different concentrations, the inhibitory effect of compounds AGAs1–3 on the mycelial growth of *F. graminearum* is significantly better than that of unmodified compound GA, indicating that compared with the unmodified complex GA, the azole-modified GA derivatives AGAs1–3 significantly enhance its antifungal activity against *F. graminearum* due to the introduction of nitrogen heterocycles. At the same time, it was demonstrated that the inhibitory effect of GA derivatives modified with azoles AGAs1–3 on *F. graminearum* was a synergistic effect of azoles and GA, which was consistent with our expectations. These results indicate that the structure of GA derivatives containing acyl imidazole, acyl pyrazole and imidazolium salts effectively enhances its antifungal activity against *F. graminearum*. This phenomenon may be attributed to the p-π conjugation between acylimidazole and acylpyrazole heterocyclic groups, which can form a large conjugated system in GA derivatives containing acyl azole structures. In addition, the lone pair electrons of the heteroatom (N) in pyrazole and imidazole increased the density of the electron cloud on the derivatives of GA, which could enhance the interaction between the compounds and its receptor, thereby improving the antifungal effect. In addition, the possible reason for AGAs–3 to have the highest antifungal activity against *F. graminearum* might be attributed to the quaternary ammonium salt structure itself.

Spore germination is a crucial step for filamentous fungi to colonize in new environments, leading to the formation of polarized growth axes. Therefore, the inhibitory activity of five compounds on spore germination was tested. Figure 3B shows the inhibitory rates of five compounds at a concentration of 0.4 mg/mL on the germination of *F. graminearum* spores. The inhibitory rates of the five compounds on *F. graminearum* spore germination ranged from 70% to 96%, indicating that they all had a certain protective effect against Fusarium head blight. The inhibitory rate of AGAs–3, the gallic acid derivative imidazolium salt, on *F. graminearum* spore germination was 95.45%, indicating that the protective effect of AGAs–3 compounds against wheat scab was significantly improved compared to GA. This might be due to the fact that compound AGAs–3 has a quaternary ammonium salt structure and exhibits a positive charge in aqueous solutions, making it easier to bind to the fungal spore cell membranes. By changing the permeability of cell membranes, making enzymes or proteins denatured, affecting cell metabolism and inhibiting their activity, AGAs–3 achieves antifungal effects [40].

**Figure 4:**
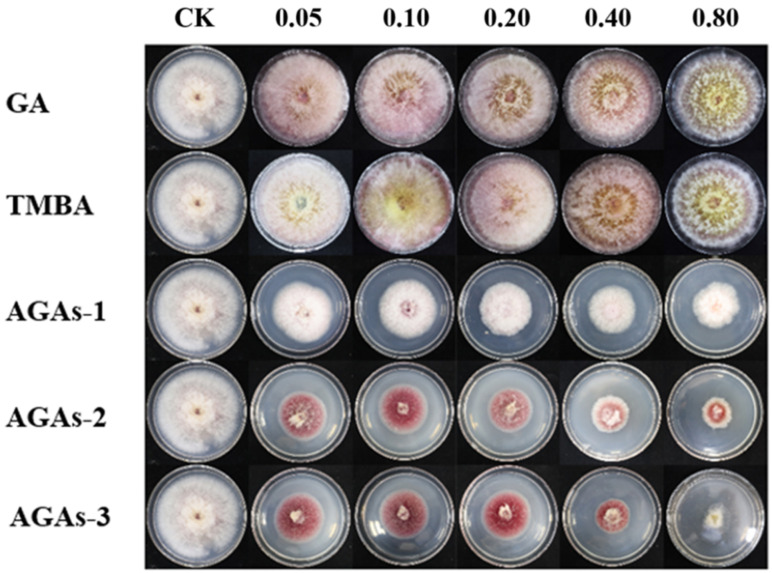
The antifungal activity of GA and its derivatives against *F. graminearum* in vitro (cultured continuously at 28 ± 1 °C for 3 days, mg/mL).

Additionally, the EC_50_ values of the five compounds (GA, TMBA, and AGAs1–3) on the growth of *F. graminearum* mycelium are shown in Table 1, which indicates that the EC_50_ of compounds AGAs1–3 is significantly lower than that of GA. Especially for AGAs–3, the EC_50_ value is 0.42 mg/mL. In addition, it can be clearly seen from the experimental photos of mycelial growth rate that compounds AGAs1–3 have a significant inhibitory effect on the growth of *F. graminearum* hyphae in a dose-dependent manner (Figure 4).

The above research results indicate that unmodified GA exhibits extremely low antifungal activity in inhibiting the growth of *F. graminearum* hyphae at different concentration levels. However, the introduction of imidazole, pyrazole, and imidazolium salt significantly enhanced the antifungal activity of GA against *F. graminearum*, indicating that the production of pharmacological groups such as acylimidazole, acylpyrazole, and imidazolium salts in GA derivatives play a crucial inhibitory role on the growth of *F. graminearum* hyphae. The possible reason for this might be, on the one hand, that the specific chemical structure of GA derivatives AGAs1–3 can affect the electron transfer process which plays an important role in its interaction with fungal cell membrane receptors. On the other hand, different mechanisms such as chemical reactions, biological effects, and physical barriers when the mycelium interacts with the GA derivatives AGAs1–3, result in potential damage to the mycelium. These mechanisms may individually or in coordination promote antifungal activity against *F. graminearum* [41].

### 2.3. The Effect of Compound AGAs–3 on Mycelial Morphology

**Figure 5:**
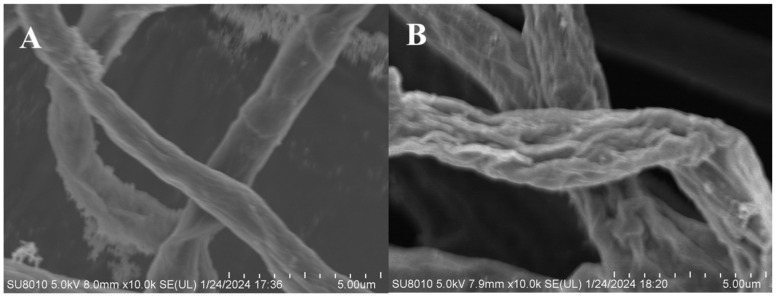
SEM of *F. graminearum* at 10.0k×. (**A**) Blank, (**B**) The surface morphology of *F. graminearum* mycelium treated by composite AGAs-3.

The morphology of *F. graminearum* mycelium was observed under scanning electron microscopy (SEM) (Figure 5). From the SEM images, we can see that the mycelium of *F. graminearum* in the blank group grows vigorously, with a good extensibility, uniform thickness, and a relatively plump and smooth surface morphology (Figure 5A). After treatment with compound AGAs–3, the surface morphology of the mycelium of *F. graminearum* underwent significant collapse, deformation, shrinkage, and varying degrees of distortion (Figure 5B).

### 2.4. Field Efficacy Trials of GA and Its Azole Derivatives against F. graminearum In Vivo

After the ascospores of *F. graminearum* spread to wheat ears, they germinate and produce hyphae when exposed to water at a certain temperature, invading the tissue of wheat ears and causing disease. The incidence rate and disease index of wheat scab disease under the treatment of GA and its derivatives TMBA and AGAs1–3 in the field test are shown in Table 2. Compared with GA, the azole-modified gallic acid derivatives AGAs–2 and AGAs–3 significantly reduced the disease incidence rate and the disease severity index is much lower than that of the blank group. The preventive effect of compound AGAs–3 is as high as 72.11%, indicating that its antifungal activity is significantly better than that of unmodified compounds, which is consistent with the results of the spore germination experiment. These results indicate that compounds AGAs–2 and AGAs–3 can serve as potential plant-based fungicides in the field of plant protection.

**Figure 6:**
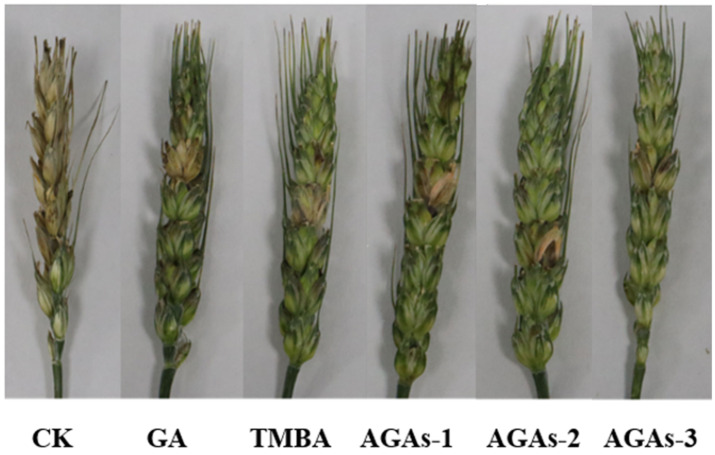
In vivo antifungal activity against *F. graminearum* under different treatments (0.4 mg/mL).

Figure 6 shows the antifungal activity of GA and its derivatives TMBA and AGAs1–3 against *F. graminearum*. In field experiments, the antifungal activity of compounds AGAs–2 and AGAs–3 against *F. graminearum* was significantly better than other compounds, indicating that these two compounds have good antifungal activity in vitro and are a promising antifungal agent for controlling crop fungal diseases.

### 2.5. Theoretical Calculation

Frontier molecular orbitals are important predictive indicators of molecular polarization and reactivity. Most chemical reactions occur by transferring electrons from the highest occupied molecular orbital (HOMO) of the donor to the lowest unoccupied molecular orbital (LUMO) of the acceptor [42]. When drug molecules interact with fungi, they may utilize electron transfer and other interactions between drug molecules and the main components of fungal cell walls, such as polysaccharides, proteins, or lipid receptors, to achieve antifungal effects.

**Figure 7:**
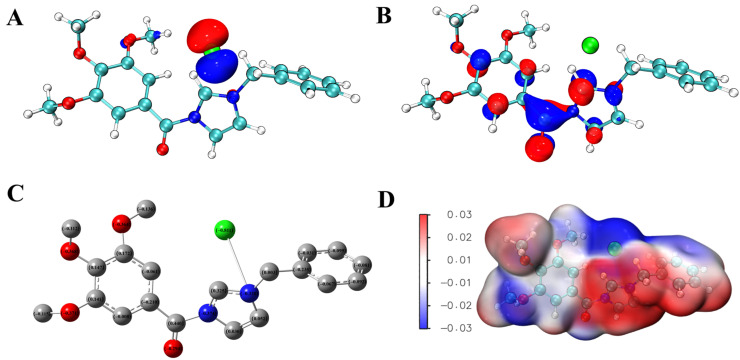
HOMO orbitals (**A**), LUMO orbitals (**B**), charge distribution (**C**), and MEP of compound AGAs–3 (**D**).

Taking the imidazolium salt AGAs–3, which has the highest inhibitory activity against *F. graminearum*, as an example, the distribution of the HOMO and LUMO orbitals of compound AGAs–3 were calculated at the same theoretical level, as shown in Figure 6. It can be observed that the HOMO of AGAs–3 is mainly located on the chlorine atom in the molecule, which is determined by the strong electron withdrawing effect of the chlorine atom (Figure 7A). The LUMO of AGAs–3 is mainly located in the region where the imidazole ring and carbonyl group are located (Figure 7B), which are the active centers that play a key role in the interaction between AGAs–3 and fungal cell membrane receptors. When it interacts with the cell membrane receptor, the chlorine atom provides electrons to the receptor, while where the imidazole ring and carbonyl group are located is an onium salt structure, and of which the other side connecting the carbonyl group is a benzene ring structure. All of these can form a large, delocalized structure, promoting the stable flow of electrons from the cell membrane receptor toward the delocalized region, and causing it to bind with the cell membrane receptor to form electron transfer complexes, breaking the original structure of the cell membrane receptor, ultimately leading to changes in the regulatory mechanism of the cell membrane receptor and achieving antifungal effects.

Combining the molecular electrostatic potential (MEP) plot can more intuitively illustrate this phenomenon. The MEP plot is related to the total electron density and is commonly used to identify regions that are prone to electrophilic and nucleophilic reactions, as well as hydrogen bonding interactions, and to define regions with local negative and positive charges within the molecule. MEP reflects the electron density and indicates the affinity between drugs and receptors. Figure 7D shows the MEP of AGAs–3, with a blue electron rich region indicating that electrons are at a high energy level and have a large number of electrons, making it easy to abandon them. Due to the strong electron withdrawing effect of Cl atoms, a large amount of negative charges are accumulated in this region. Therefore, when compound AGAs–3 interacts with the protein or enzyme of the receptor, this part mainly provides electrons, forming an electron transfer process, thereby achieving antifungal effects. The imidazole ring and carbonyl structure of compound AGAs–3 are shown in red on the MEP diagram, indicating that the accumulation of a significant amount of positive charge in this region, which mainly receives electrons provided by the receptor when interacting with it.

## 3. Materials and Experimental Methods

### 3.1. Materials

Silicone (100–200 mesh/200–300 mesh, Qingdao Gulf Fine Chemical Co., Ltd., Qingdao, China), dimethyl sulfate (AR, Chengdu Huaxia Chemical Reagent Co., Ltd., Chengdu, China), imidazole (AR, Shanghai Darui Fine Chemicals Co., Ltd., Shanghai, China), pyrazole (AR, Shanghai Darui Fine Chemicals Co., Ltd., Shanghai, China), benzyl chloride (AR, Aladdin Reagent Co., Ltd., Shanghai, China), 1-ethyl-(3-dimethylaminopropyl) carbodiimide (1-(3-dimethylaminopropyl)-3-ethylcarbodiimide (EDC) (AR, Tianjin heowns bio-chemical Technology Co., Ltd., Tianjin, China), 1-hydroxybenzotriazole (HOBT) (AR, Tianjin heowns bio-chemical Technology Co., Ltd., Tianjin, China), concentrated hydrochloric acid, sodium hydroxide, anhydrous sodium sulfate (Na_2_SO_4_), anhydrous ether, anhydrous ethanol, petroleum ether, and ethyl acetate analytical purity agents were purchased from Tianjin Oubokai Chemical Co., Ltd. (Tianjin, China), while dichloromethane (CH_2_Cl_2_) and acetonitrile chromatographic purity agents were obtained from Thermo Fisher Scientific Technology Co., Ltd. (Waltham, MA, USA), and the experimental water was ultrapure water.

### 3.2. Preparation of GA and Its Derivatives

#### 3.2.1. Extraction and Purification of GA from Chestnut Leaves

Abandoned chestnut leaves collected from the experimental station of the Horticultural Science and Technology College of Hebei Normal University of Science and Technology were placed in a cool and ventilated place to dry and were crushed to obtain dried chestnut leaf powder. The crude extract of chestnut leaves was extracted with 95% ethanol at 60 °C, and then extracted three times with ethyl acetate. The organic phase was combined and vacuum rotary dried. Using 200–300 mesh silica gel dry method for sample loading, gradient elution was carried out using petroleum ether and ethyl acetate with polarity from small to large as eluents to obtain different components. Finally, the ethyl acetate layer component was purified by methods such as recrystallization and liquid phase preparation chromatography to obtain the target compound GA. ^1^H NMR (600 MHz, MeOD) *δ* 7.06 (s, 2H, -C_6_*H*_2_-). ^13^C NMR (150 MHz, MeOD) *δ* 108.92, 120.57, 138.19, 145.00 (-*C*_6_H_2_-), 168.98 (-*C*=O). HR-ESI(-)-MS(*m*/*z*): 169.0144 (Calcd. for C_7_H_5_O_5_: 169.0137 [M − H]^−^, 100%).

#### 3.2.2. Synthesis of Azole-Modified Gallic Acid Derivatives (AGAs1–3)

The synthesis of AGAs1–3 requires protecting the three phenolic hydroxyl groups of GA to obtain the intermediate 3,4,5-trimethoxybenzoic acid (TMBA). TMBA was prepared according to the reporting method with slight modifications [43]. Firstly, placing 100 mL of aqueous solution containing 50 mmol of gallic acid in a 250 mL four-necked bottle, slowly adding 120 mmol of sodium hydroxide and 120 mmol of dimethyl sulfate under nitrogen protection, stirring for 10 min, then adding 120 mmol of dimethyl sulfate again and stirring for 1 h. The entire process was controlled below 35 °C. Finally, 120 mmol sodium hydroxide was added and refluxed for 5 h. After cooling the reaction solution down to room temperature, it was acidified with concentrated hydrochloric acid to pH = 2, filtered to obtain a white solid, recrystallized with water, and dried. Finally, 8.7 g intermediate compound TMBA was obtained. ^1^H NMR (600 MHz, CDCl_3_) *δ* 3.93(s, 6H, -OC*H*_3_), 3.94(s, 3H, -OC*H*_3_), 7.38(s, 2H, -C_6_*H*_2_-). ^13^C NMR (150 MHz, CDCl_3_) *δ* 56.27(-O*C*H_3_), 60.98 (-O*C*H_3_), 107.45, 124.05, 143.03, 153.00 (-*C*_6_H_2_-), 171.45 (-*C*=O). HR-ESI(-)-MS(*m*/*z*): 211.0615 (Calcd. For C_10_H_11_O_5_: 211.0606 [M − H]^−^, 100%).

Synthesis of compound AGAs–1 began with adding 1 mmol TMBA and 1 mmol imidazole to a CH_2_Cl_2_ solvent containing 1.1 mmol 1-ethyl-(3-dimethylaminopropyl)-3-ethylcarbodiimide (EDC) and 1.1 mmol 1-hydroxybenzotriazole (HOBT), and stirring at room temperature for 24 h [27]. Vacuum distillation, extracted three times with ethyl acetate (3 × 30 mL), combined with organic phases, dried with anhydrous Na_2_SO_4_ for 30 min, filtered, and subjected to vacuum distillation to obtain the crude product white solid. The crude product was separated and purified using column chromatography, with 100–200 mesh silica gel as the stationary phase and petroleum ether: ethyl acetate = 4:1 (*V*:*V*) as the eluent. After purification, the target compound AGAs–1 was obtained. ^1^H NMR (600 MHz, CDCl_3_) *δ* 3.97 (s, 6H, -OC*H*_3_), 4.01 (s, 3H, -OC*H*_3_), 7.46–7.50 (dd, 1H, imidazole 4-*H*), 7.52 (s, 2H,-C_6_*H*_2_-), 7.56–7.59 (t, *J* = 7.4 Hz, 1H, imidazole 5-*H*), 8.12–8.13 (d, *J* = 8.5 Hz, 1H, imidazole 2-*H*). ^13^C NMR (150 MHz, CDCl_3_) *δ* 56.49 (-O*C*H_3_), 61.21 (-O*C*H_3_), 108.01, 108.42, 119.13, 120.65, 124.94, 128.84, 143.60, 144.46 (-*C*_6_H_2_-, imidazole 4-*C*, 5-*C*), 153.42 (imidazole 2-*C*), 162.46 (-*C*=O). HR-ESI(+)-MS(*m*/*z*): 285.0839 (Calcd. For C_13_H_14_N_2_O_4_Na: 285.0846 [M + Na]^+^, 100%).

The synthesis method of AGAs–2 was the same as that of AGAs–1, with Im replaced by Py. ^1^H NMR (600 MHz, CDCl_3_) *δ* 3.92 (s, 6H, -OC*H*_3_), 3.951 (s, 3H, -OC*H*_3_), 6.54 (s, 1H, pyrazole 4-*H*), 7.50 (s, 2H,-C_6_*H*_2_-), 7.81 (s, pyrazole 3-*H*), 8.45 (d, *J* = 2.5 Hz, 1H, pyrazole 5-*H*). ^13^C NMR (150 MHz, CDCl_3_) *δ* 56.32 (-O*C*H_3_), 60.96 (-O*C*H_3_), 109.30, 109.52, 120.64, 124.09, 126.02, 128.80, 130.80, 142.62, 144.46 (-*C*_6_H_2_-, pyrazole 3-*C*, 4-*C*), 152.66 (pyrazole 5-*C*), 165.52 (-*C*=O). HR-ESI(+)-MS(*m*/*z*): 285.0841 (Calcd. For C_13_H_14_N_2_O_4_Na: 285.0846 [M + Na]^+^, 100%).

Synthesis of compound AGAs–3 began with adding 1.0 mmol of compound AGAs–1 and 1.1 mmol of benzyl chloride to a round bottom flask containing 30 mL of acetonitrile solvent, heat and reflux at 80 °C for 24 h, cool to room temperature, distill under reduced pressure to obtain crude product, then wash with anhydrous ether and dry to obtain the target compound AGAs–3. ^1^H NMR (600 MHz, CDCl_3_) *δ* 3.97 (s, 6H, -OCH_3_), 4.01 (s, 3H, -OCH_3_), 4.60 (s, 2H, -CH_2_C_6_H_5_), 7.32–7.40 (m, 5H, -CH_2_C_6_H_5_), 7.45–7.50 (m, 1H, imidazole -*H*), 7.52 (s, 2H, -C_6_H_2_-), 7.56–7.58 (t, *J* = 7.8 Hz, 1H, imidazole 5-*H*), 8.11–8.13 (d, *J* = 8.4 Hz, 1H, imidazole 2-*H*). ^13^C NMR (150 MHz, CDCl_3_) *δ* 46.31 (-CH_2_C_6_H_5_), 56.48 (-OCH_3_), 61.20 (-OCH_3_), 108.03, 108.42, 119.12, 120.63, 124.93, 128.424, 128.60, 128.76, 128.84,137.49, 143.60, 144.50 (-C_6_H_2_-, -CH_2_C_6_H_5_, imidazole 4-*C,* 5-*C*), 153.42 (imidazole 2-*C*), 162.46 (-*C*=O). HR-ESI(+)-MS: 353.1479 (Calcd. for C_20_H_21_N_2_O_4_Cl: 353.1496 [M − Cl]^+^, 100%).

### 3.3. Characterization

The structures of all the final products were identified and confirmed by nuclear magnetic resonance spectroscopy (NMR, 600 MHz, Bruker, Germany) and high resolution mass spectroscopy (HR-ESI-MS, LTQ Orbitrap XL, Thermo Fisher, Waltham, MA, USA). The mycelial morphology of *F. graminearum* was observed with scanning electron microscopy (SEM, SU8010, Hitachi, Tokyo, Japan).

### 3.4. Mycelial Growth Rate Experiment

We mixed 444.4 g of potatoes, 35.2 g of agar, and 35.2 g of sugar in 450 mL of water and heated it to boiling to produce PDA. Then, we divided the PDA into several conical bottles and sealed them at 121 °C for sterilization about 30 min before using.

Three replicates were set for each concentration in this experiment, and PDA was used as a blank control after equal volume sterilization. We weighed 120 mg of GA, added 20 mL of deionized water, and sonicated for 1 h until all GA was dissolved. We mixed it with PDA to prepare toxic culture media with concentrations of 0.05 mg/mL, 0.1 mg/mL, 0.2 mg/mL, 0.4 mg/mL, and 0.8 mg/mL. Then, we inoculated *F. graminearum* onto a toxic culture medium and cultured continuously for three days at a temperature of 28 ± 1 °C. All in vitro antifungal test methods for drugs were carried out according to the above procedure.

We used the cross-over method to determine the colony diameter, calculated the average diameter size, and calculated the antibacterial rate of the drug according to Formula (1) [44,45]:

CD (%) = (dc − dt)/(dc − 1) × 100%(1)

where dt is the average colony diameter of the treatment group, and dc is the average colony diameter of the blank control group.

### 3.5. In Vitro Antibacterial Activity Testing of Spore Germination

We made 100 μL spore suspension (3 × 10^−7^ spore mL^−1^) and 100 μL solution containing AGAs–3 to prepare a drug spore suspension with a concentration of 0.4 mg/mL. Then, we took 30 μL of the above suspension and placed it on a sterile concave glass slide. After completely dark cultivation at 28 ± 1 °C for 5 h, we measured the spore germination rate and at least 200 spores per treatment.

We maintained 100% humidity during germination. Formula (2) was used for calculating spore germination rate as follows:

spore germination rate (%) = (number of germinated spores)/(total number of spores) × 100(2)

### 3.6. Methods of Sample Preparation for Scanning Electron Microscopy 

The cultured hyphae was fixed in a refrigerator at 4 °C with 2.5% pH = 6.8 glutaraldehyde for 6 h, and then rinsed with 0.1 mol/L pH = 6.8 phosphate buffer solution, followed by gradient dehydration and displacement with different concentrations of ethanol and tert-butanol. Finally, the sample was freeze-dried and sprayed with a layer of 1500 nm thick gold film.

### 3.7. Antifungal Activity of GA and Its Derivatives In Vivo

Field efficacy trials of GA and its azole derivatives (GA, TMBA, AGAs1–3) against wheat scab were conducted at the experimental station of Hebei Normal University of Science and Technology. Wheat seeds (Shaochuan 02–1) were purchased from Hebei Academy of Agricultural and Forestry Sciences. The antifungal activity of compounds GA, TMBA, and AGAs1–3 against *F. graminearum* was determined using the single-sided small flower inoculation method [46]. During the early flowering stage of wheat, spore suspension was injected into a single side small flower of a spike (*F. graminearum* spore suspension was mixed with compounds GA, TMBA, and AGAs1–3 to form a 0.4 mg/mL inoculum, and sterile water was used as a negative control). We used self-sealing bags to seal wheat ears for three days to maintain humidity and promote disease development. Seven days after 100 spikelets were inoculated with each drug, the incidence rate and disease index of spikelets were counted [47]. Seven days after onset, the incidence rate of disease and disease index were visually assessed using a severity level of 0–100%.

Classification method:

Level 0, Whole ear disease-free;

Level 1, the area of withered panicles accounts for less than 1/4 of the total panicle area;

Level 3, the area of withered panicles accounts for 1/4–1/2 of the total panicle area;

Level 5, the area of withered panicles accounts for 1/2–3/4 of the total panicle area;

Level 7, the area of withered panicles accounts for more than 3/4 of the total panicle area.

We calculated the diseased ear rate according to Formula (3),

D = N_d_/N × 100 (3)

where D is the diseased panicle rate, in percentage, and N_d_ is the number of diseased ears, N is the total number of surveys.

The disease severity index was calculated according to Formula (4),

X = ∑ (N_i_ × i)/N × 7 × 100 (4)

where i is the severity levels of the condition.

### 3.8. Theoretical Calculation

Quantum chemical calculations were completed using the Gaussian 09 D.01 program [48]. Structural optimization was completed using B3LYP [49,50] functional combined with 6-31G (d, p) basis set [51]. The molecular surface electrostatic potential map and frontier molecular orbital contour map were rendered using the VMD 1.9.3 program [52], and the VMD rendered files were obtained from Multiwfn 3.8(dev) code [53].

## 4. Conclusions

Reasonable use of pesticides is an important measure to prevent and control wheat scab to improve the yield and quality of wheat. This article successfully synthesized three new compounds using natural polyphenol GA as raw material, and identified their structures through NMR and HRMS. The antifungal ability of GA and its derivatives against *F. graminearum* was measured at different concentrations using *F. graminearum* as a model strain. The results showed that compared with GA, AGAs1–3 exhibited significant antifungal activity against *F. graminearum* in a dose-dependent manner. The introduction of imidazolium salt structure can significantly inhibit the growth of *F. graminearum* with an EC_50_ of 0.42 mg/mL and an inhibition rate of 73.5% on the mycelial growth of *F. graminearum*. When 0.4 mg/mL of AGAs–3 was applied, the control effect of wheat FHB in the field reached 72.11%, significantly reducing the degree of infection of *F. graminearum* on wheat, demonstrating a good control effect. Therefore, GA derivatives with imidazolium salt structure can provide a theoretical basis for the development of green and environmentally friendly fungicides for plant pathogenic fungi.

## Figures and Tables

**Table 1:** The EC_50_ values of compounds GA, TMBA, and AGAs1–3 in inhibiting the mycelial growth of *F. graminearum*.

Compound	Slope ± Standard Error	EC_50_ (mg/mL) (95% Confidence Interval)
GA	2.47 ± 1.42	10.62 (0.10–1089.02)
TMBA	2.45 ± 1.42	10.92 (0.10–1153.72)
AGAs–1	1.07 ± 0.04	0.64 (0.57–0.72)
AGAs–2	0.33 ± 0.03	0.49 (0.39–0.61)
AGAs–3	1.40 ± 0.26	0.42 (0.27–0.66)

**Table 2:** Field control effects of compounds on *F. graminearum*.

Treatment	Number of Surveyed Plants	Disease Incidence (%)	Disease Severity Index	Control Efficacy (%)
CK	94	92.55	76.064	-
GA	95	95.23	30.015	55.13
TMBA	88	94.32	29.545	56.73
AGAs–1	94	90.43	31.383	58.74
AGAs–2	95	74.74	23.684	68.86
AGAs–3	99	78.79	21.212	72.11

## Data Availability

Data are contained within the article and Supplementary Materials.

## Supplementary Materials

The following supporting information can be downloaded at: https://www.mdpi.com/article/10.3390/molecules29091996/s1. Figure S1: The ^1^H NMR spectrum of compound GA; Figure S2: The ^13^C NMR spectrum of compound GA; Figure S3: The ^1^H NMR spectrum of compound TMBA; Figure S4: The ^13^C NMR spectrum of compound TMBA; Figure S5: The ^1^H NMR spectrum of compound AGAs-1; Figure S6: The ^13^C NMR spectrum of compound AGAs-2; Figure S7: The ^1^H NMR spectrum of compound AGAs-1; Figure S8: The ^13^C NMR spectrum of compound AGAs-2; Figure S9: The ^1^H NMR spectrum of compound AGAs-3; Figure S10: The ^13^C NMR spectrum of compound AGAs-3; Figure S11: The HR-ESI-MS spectrum of compound GA; Figure S12: The HR-ESI-MS spectrum of compound TMBA; Figure S13: The HR-ESI-MS spectrum of compound AGAs–1; Figure S14: The HR-ESI-MS spectrum of compound AGAs–2; Figure S15: The HR-ESI-MS spectrum of compound AGAs–3.
