# Supplementary material for: Study on the Antifungal Activity of Gallic Acid and Its Azole Derivatives against Fusarium graminearum
Source: Molecules. 2024 Apr 26;29(9):1996. doi: 10.3390/molecules29091996 (PMC11085095; doi:10.3390/molecules29091996)
Supplement: Supplementary file 1 [file molecules-29-01996-s001.zip › molecules-2988001-supplementary.pdf]

Study on the Antifungal Activity of Gallic Acid and Its Azole Derivatives against *Fusarium graminearum*

Yilin Zheng [1,2], Yuqi Geng [1,2], Wenlong Hou [1,3], Zhe Li [4], Caihong Cheng [1,3,*], Xiuping Wang [3,4] and Yuedong Yang [1,2]

1 Hebei Key Laboratory of Active Components and Functions in Natural Products, Hebei Normal University of Science and Technology, Qinhuangdao 066004, China; 17333546871@163.com (Y.Z.); 17531372375@163.com (Y.G.); wenlonghou@126.com (W.H.); kycyyd@126.com (Y.Y.)
2 College of Chemical Engineering, Hebei Normal University of Science and Technology, Qinhuangdao 066004, China
3 Analysis and Testing Center, Hebei Normal University of Science and Technology, Qinhuangdao 066004, China; wangxiuping0721@163.com
4 Hebei Key Laboratory of Crop Stress Biology, College of Agronomy and Biotechnology, Hebei Normal University of Science and Technology, Qinhuangdao 066000, China; zheli2007@163.com
* Correspondence: cch20059@126.com

The NMR and HR-ESI-MS spectrum of compouds GA, TBMA and AGAs1-3 are as follows:

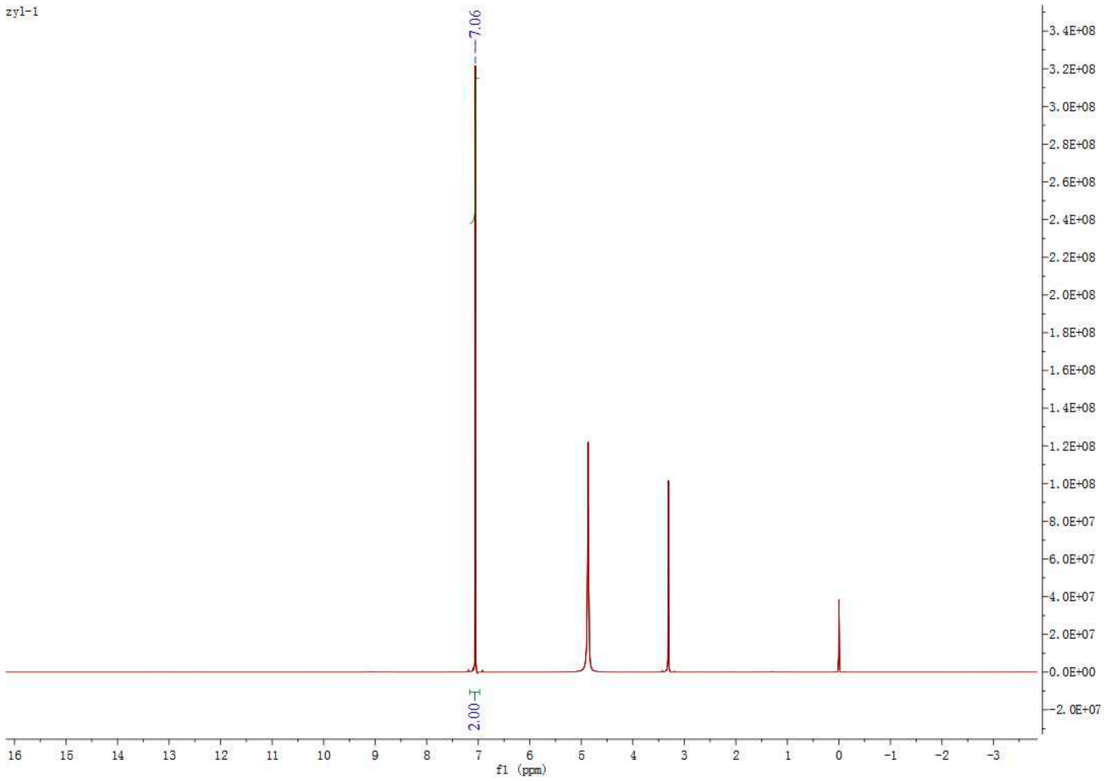

Figure S1. The ^{1}H NMR spectrum of compound GA

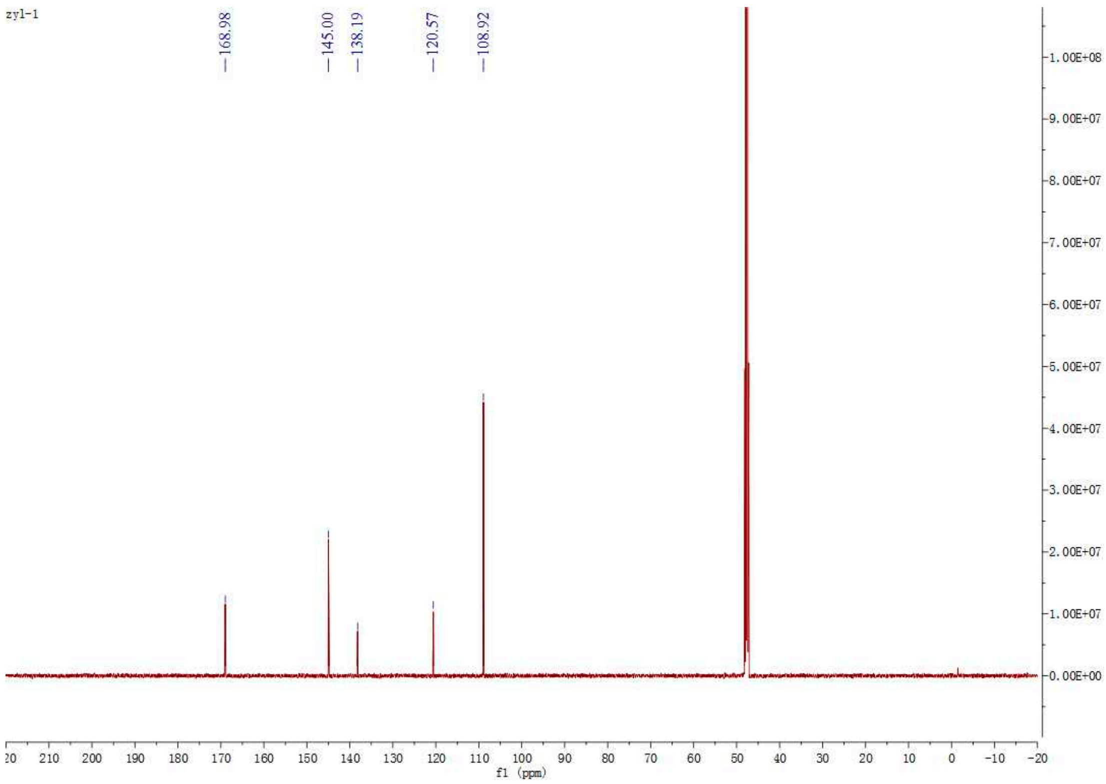

Figure S2. The ^{13}C NMR spectrum of compound GA

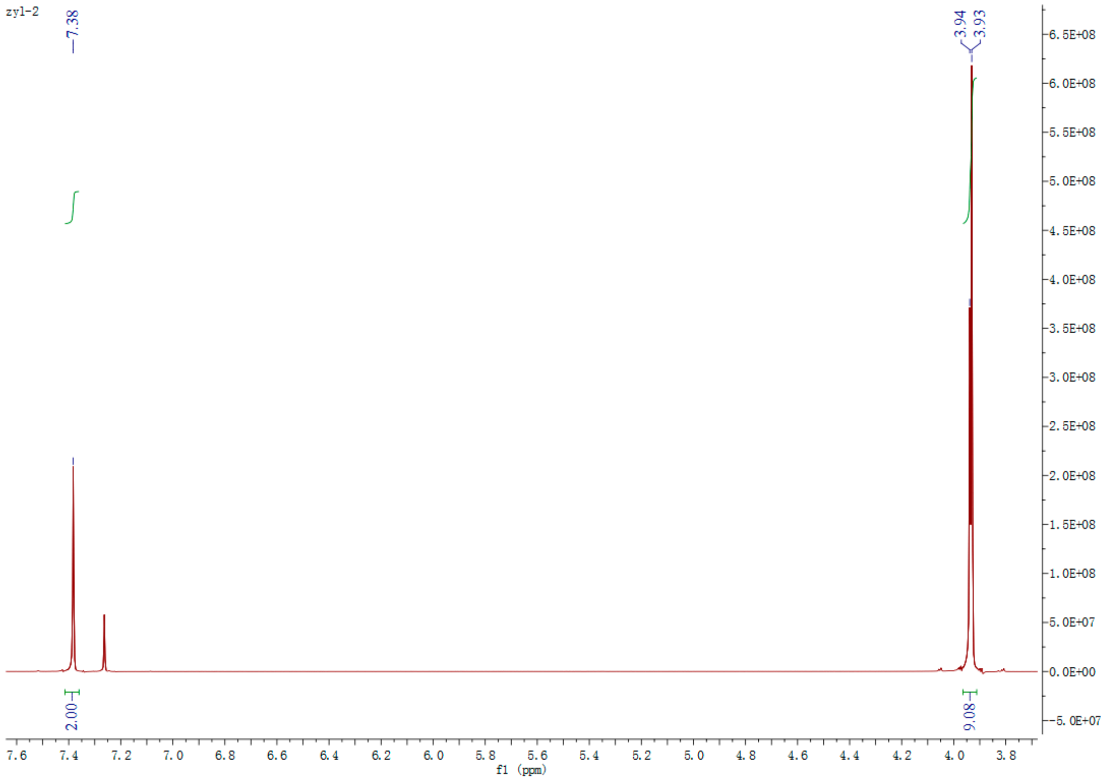

Figure S3. The ^{1}H NMR spectrum of compound TMBA

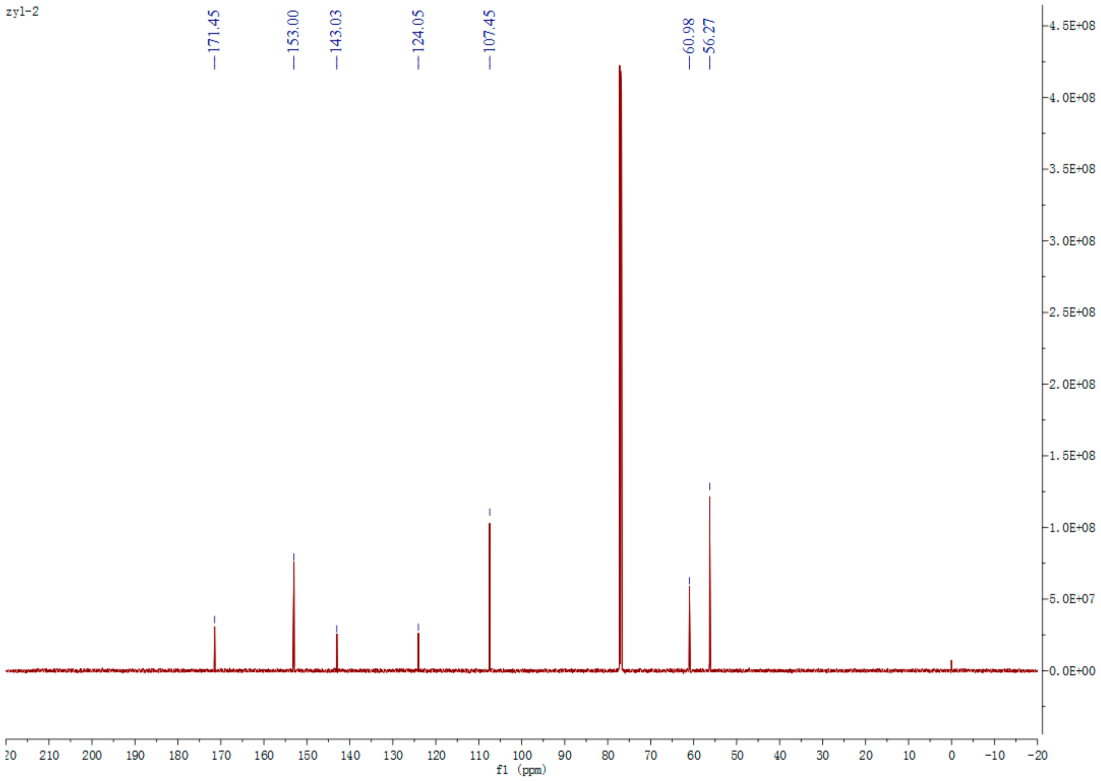

Figure S4. The ^{13}C NMR spectrum of compound TMBA

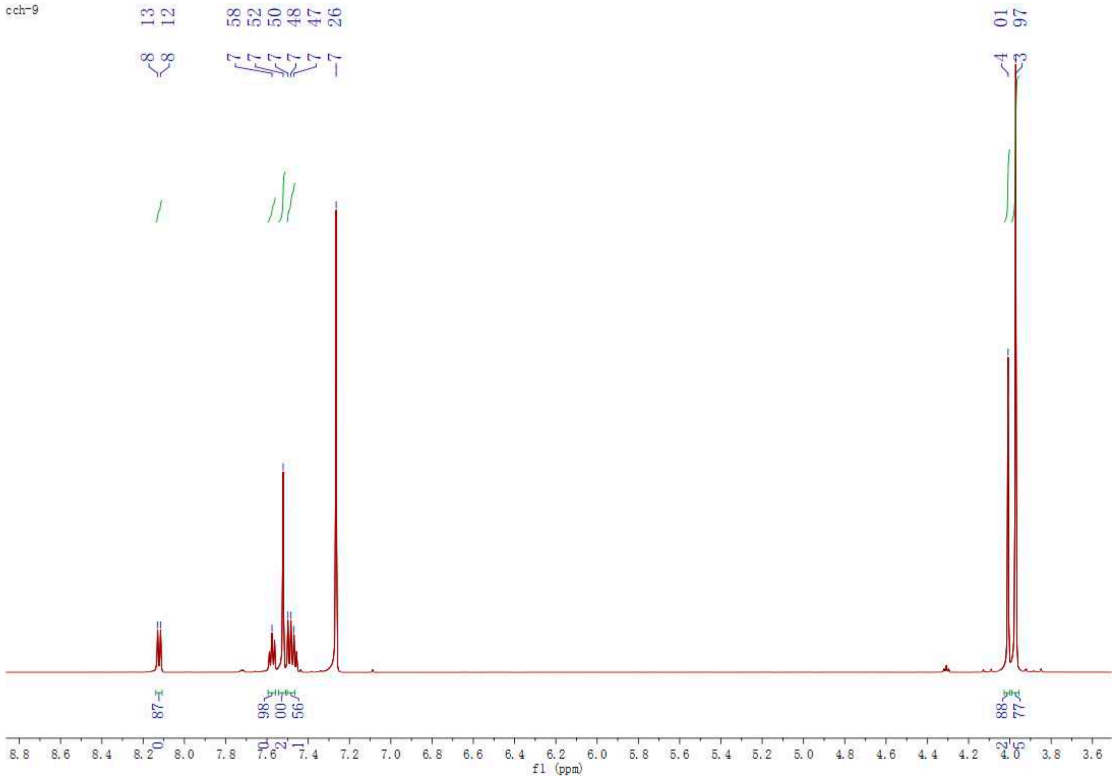

Figure S5. The ^{1}H NMR spectrum of compound AGAs-1

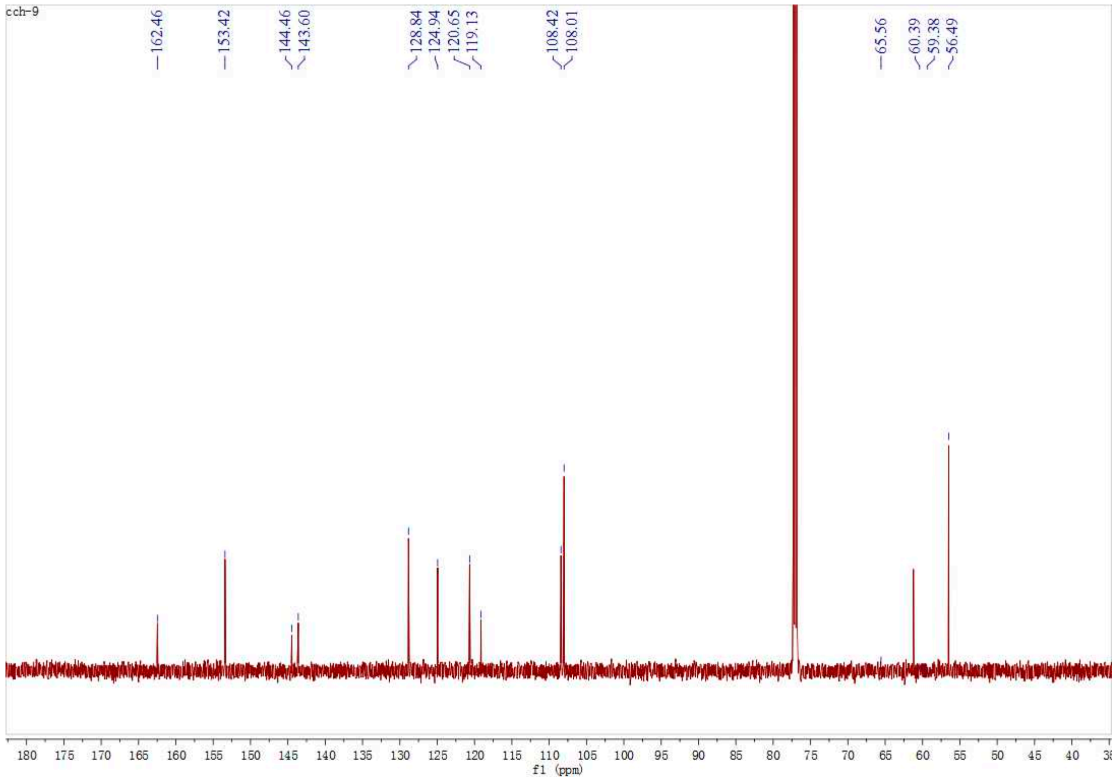

Figure S6. The ^{13}C NMR spectrum of compound AGAs-1

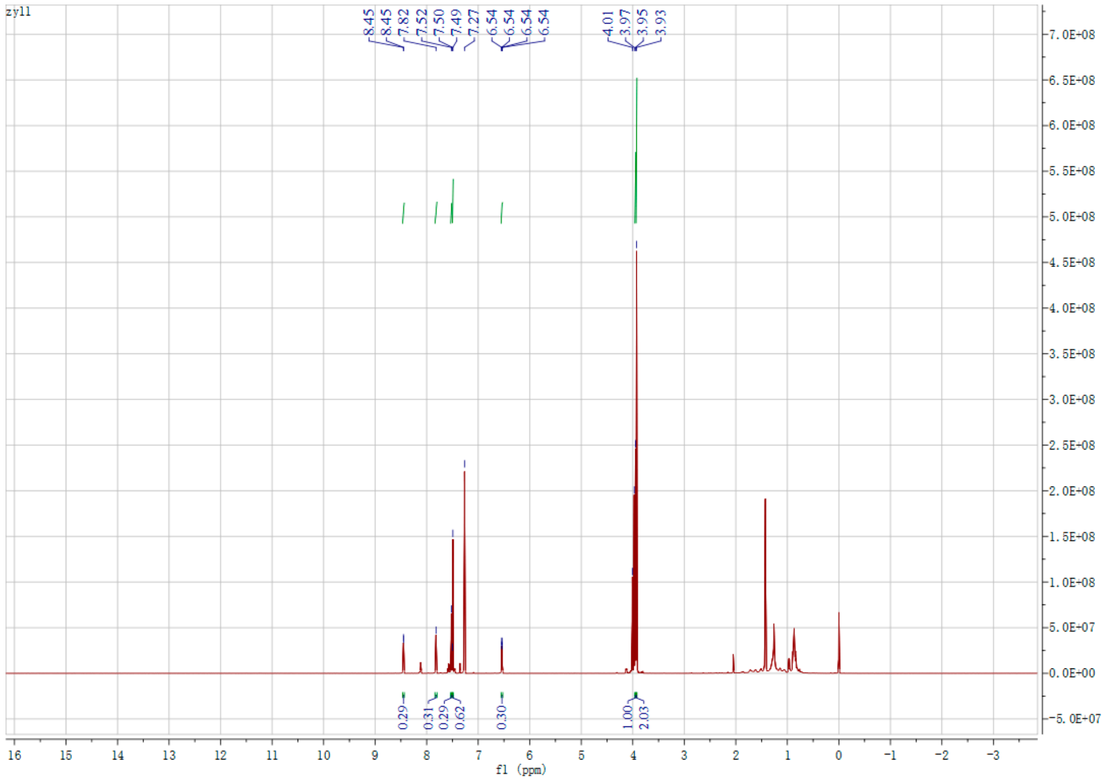

Figure S7. The ^{1}H NMR spectrum of compound AGAs-2

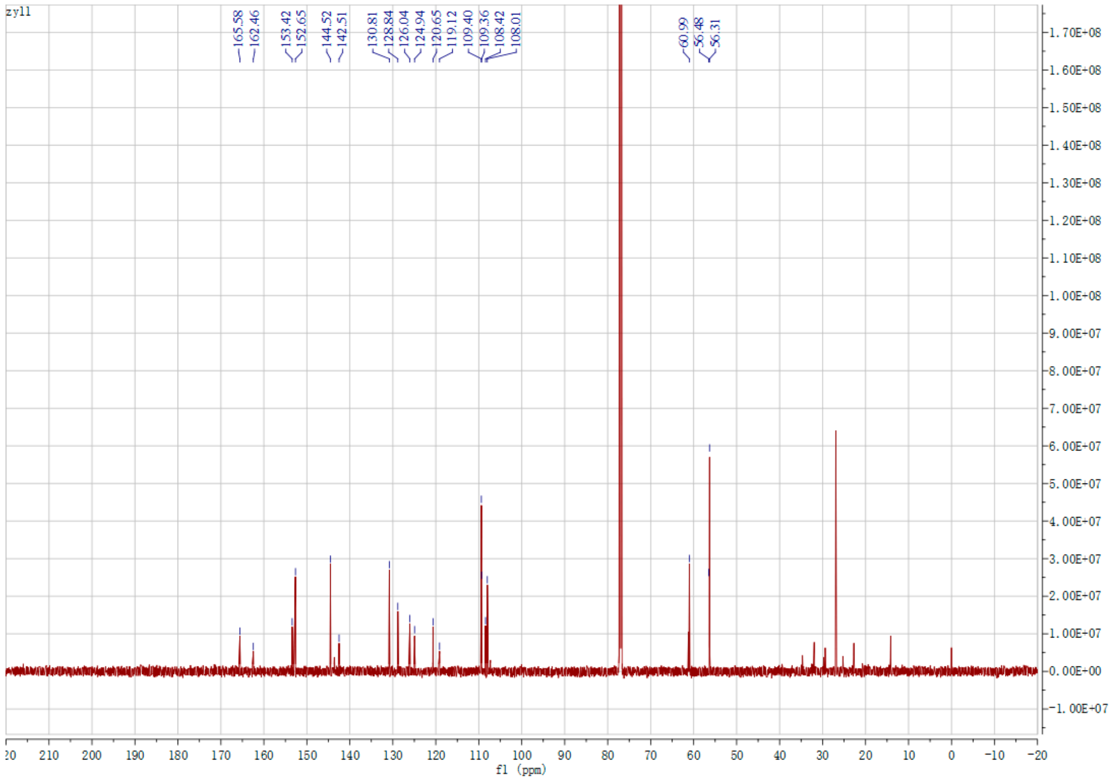

Figure S8. The ^{13}C NMR spectrum of compound AGAs-2

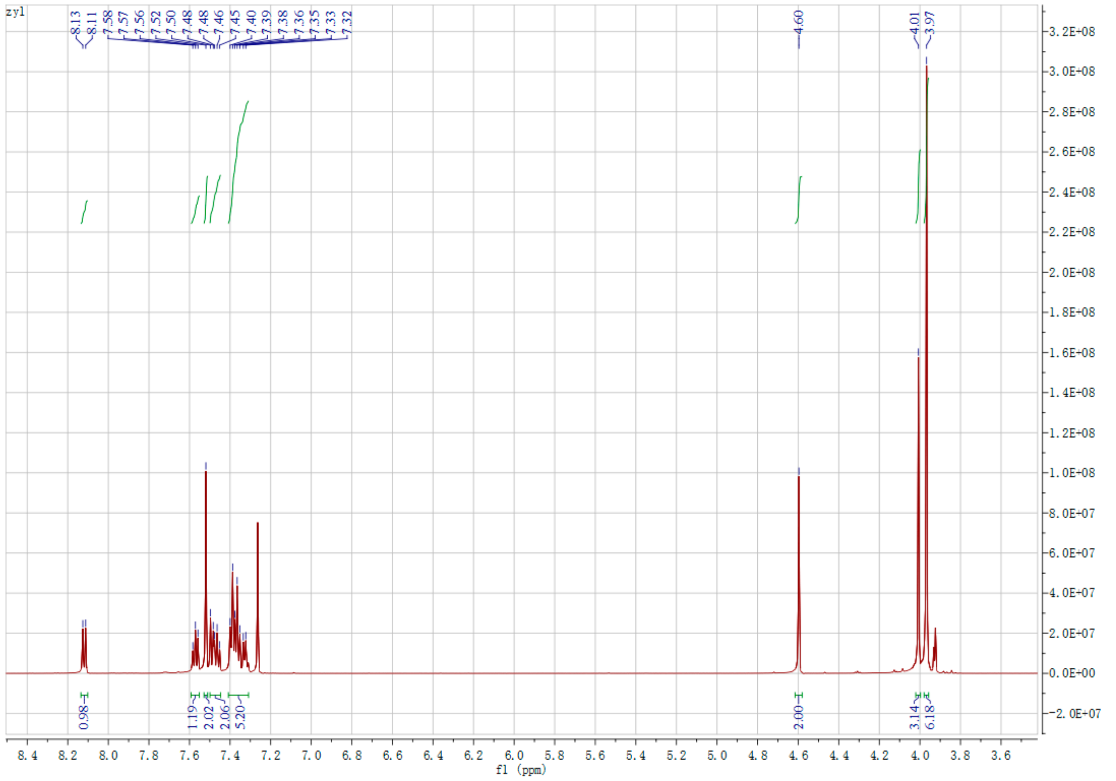

Figure S9. The ^{1}H NMR spectrum of compound AGAs-3

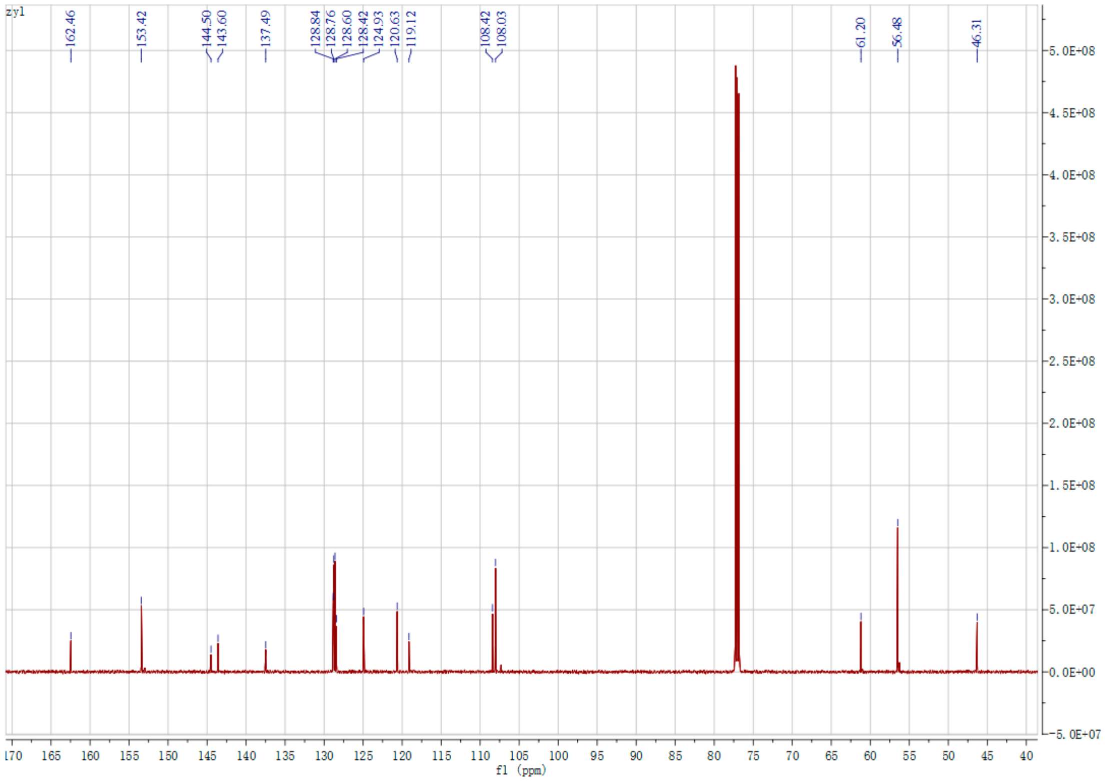

Figure S10. The ^{13}C NMR spectrum of compound AGAs-3

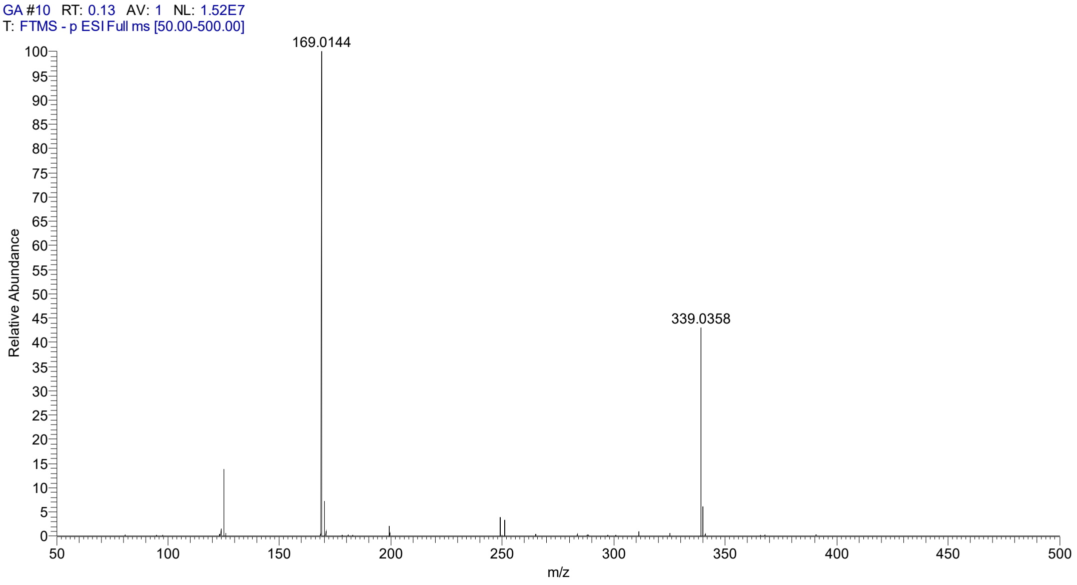

Figure S11. The HR-ESI-MS spectrum of compound GA

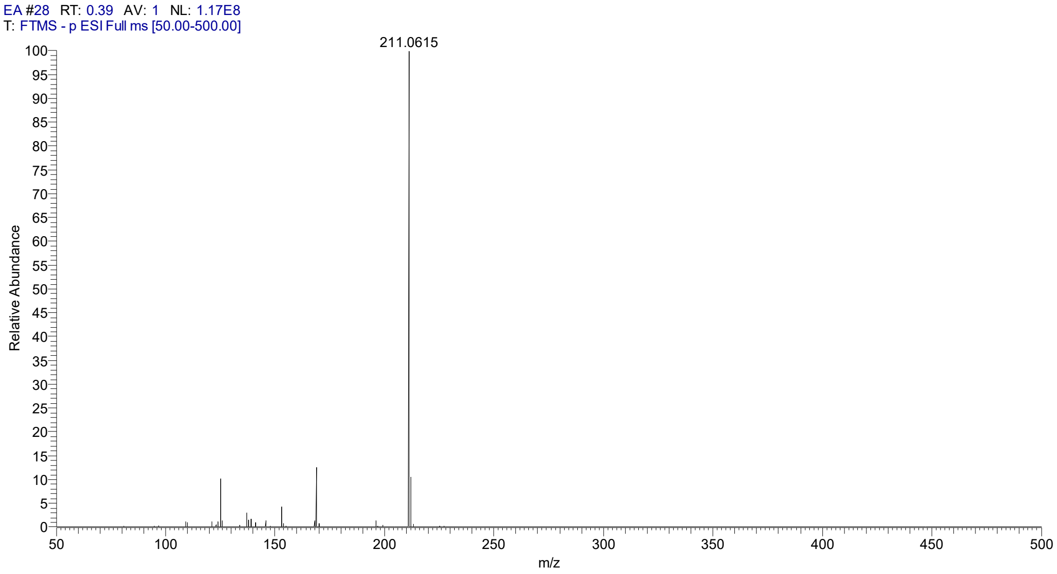

Figure S12. The HR-ESI-MS spectrum of compound TMBA

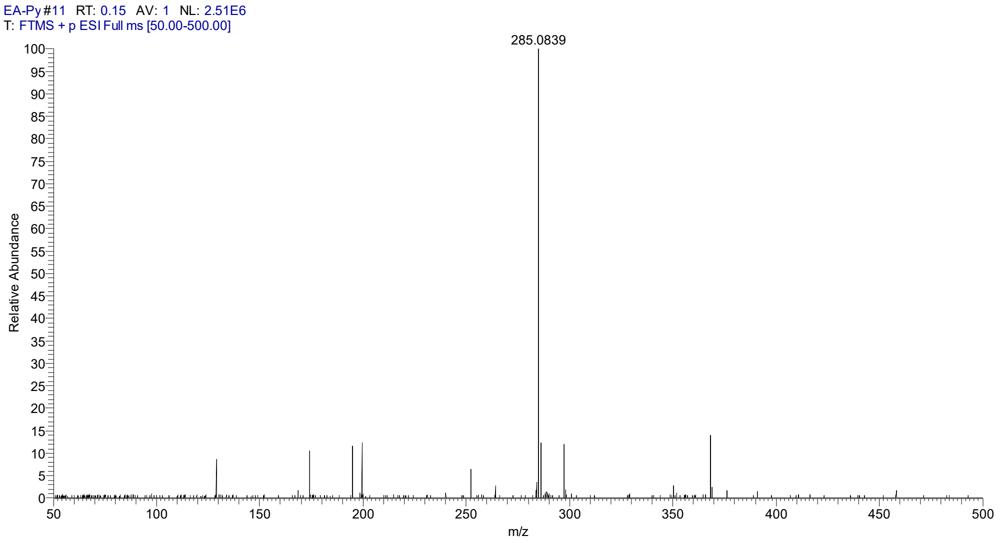

Figure S13. The HR-ESI-MS spectrum of compound AGAs-1

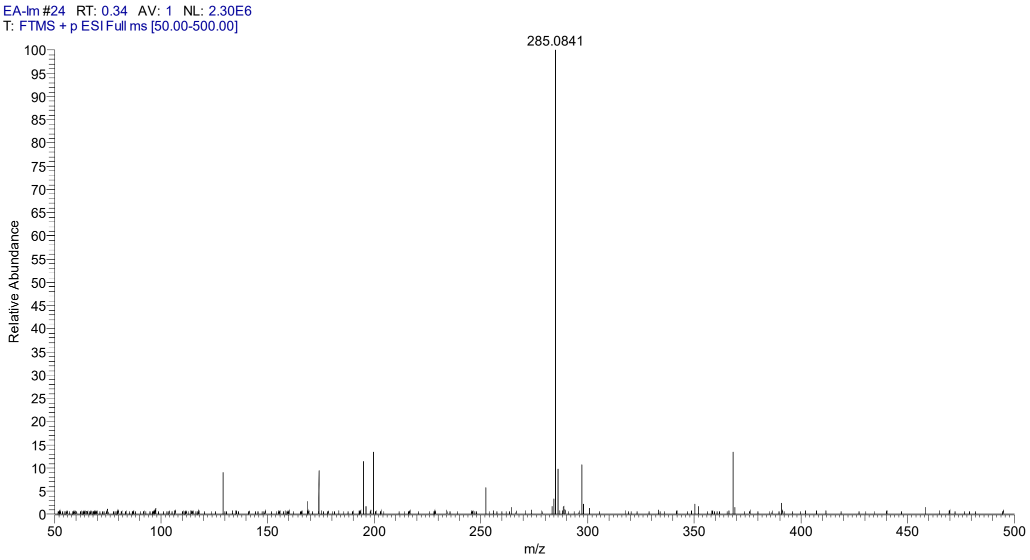

Figure S14. The HR-ESI-MS spectrum of compound AGAs-2

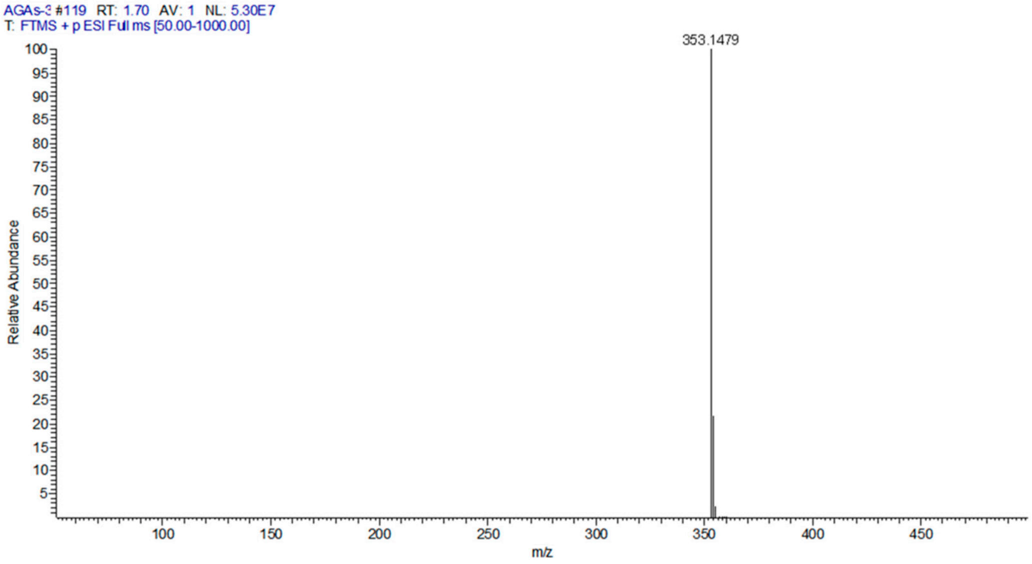

Figure S15. The HR-ESI-MS spectrum of compound AGAs-3
